# Supplementary material for: Linking aberrant chromatin features in chronic lymphocytic leukemia to transcription factor networks
Source: Mol Syst Biol. 2019 May 22;15(5):e8339. doi: 10.15252/msb.20188339 (PMC6529931; doi:10.15252/msb.20188339)
Supplement: Supplementary file 1 — Appendix [file MSB-15-e8339-s001.pdf]

Appendix

Linking aberrant chromatin features in chronic lymphocytic leukemia to transcription factor networks

Jan-Philipp Mallm, Murat Iskar, Naveed Ishaque, Lara C. Klett, Sabrina J. Kugler, Jose M. Muino, Vladimir B. Teif, Alexandra M. Poos, Sebastian Großmann, Fabian Erdel, Daniele Tavernari, Sandra D. Koser, Sabrina Schumacher, Benedikt Brors, Rainer König, Daniel Remondini, Martin Vingron, Stephan Stilgenbauer, Peter Lichter, Marc Zapatka, Daniel Mertens and Karsten Rippe

Appendix inventory

Appendix tables

Table S1. Overview of patients and healthy donors.
Table S2. Deregulated transcription factors linked to aberrant chromatin features in CLL.
Table S3. Comparison of EBF1 ChIP-seq data sets.
Table S4. Antibodies used in this study.
Table S5. ENCODE transcription factor binding site chromatin state enrichment.
Table S6. Inventory of appendix data sets.
Table S7. Data analysis software.

Appendix figures

Figure S1. Data quality assessment and analysis workflow.
Figure S2. Characterization of CLL-specific DNA methylation patterns.
Figure S3. Alternative promoter usage in CLL cells.
Figure S4. Response to CLL cells to HDAC inhibition.
Figure S5. TF binding motif analysis at enhancers predicted by additional annotations.
Figure S6. Single cell ATAC-seq analysis.
Figure S7. CLL TF network with linked deregulated chromatin modifiers and targets.
Figure S8. Analysis of EBF1 binding by ChIP-seq.

Appendix data sets

Additional data sets on samples and the analysis results derived from the different sequencing readouts are provided as separate files in Microsoft Excel format and for data set EV14 in the file format of the cytoscape network viewer (cytoscape.org). An inventory for these data sets is given in Supplementary Table S7.

Appendix references

Appendix Table S1. Overview of patients and healthy donors.

**Age (years)**			
CLL patients	Median	62	
	Range	47-79	
Healthy donors	Median	57	
	Range	51-70	
**Female**		**Number**	**%**
CLL patients		7	30
Healthy donors		31	41
**Leukocyte count (10^9/liter)**			
CLL patients	Median	101.4	
	Range	37.5 - 280.6	
**Stage**		**Number**	**%**
CLL patients	A	12	52
	B	6	26
	C	2	9
	n.a.	3	13
**Aberration**		**Number**	**%**
CLL patients	del13q	16	80
	del13q single	13	65
**IgV(H) mutational status**		**Number**	**%**
CLL patients	mutated	14	61
	unmutated	9	39

Healthy donor samples were age-matched to the CLL cohort with a median age of 57 years, compared to 62 years median age of CLL patients. 30% of CLL patients and 41% of healthy donors were female. High leukocyte counts with a median cell number of $99.6 \cdot 10^9$/liter in CLL patients reflect the result of the watch-and-wait treatment strategy of these asymptomatic patients, 52% of them classified in Binet stage A, 26% as stage B and 9% as stage C. Most patients had beneficial genetic characteristics with 70% harboring the favorable 13q chromosomal deletion and 61% a hypermutated IgV(H) status. These latter two features are associated with increased survival rates. For patient samples CLL20-22 the Binet stage and 13q status were not available.

Appendix Table S2. Deregulated transcription factors linked to aberrant CLL chromatin features.

Binding motif name in Homer [a]	Binding motif sequence logo	Predicted TFs binding to motif [b]	Site[c]	Signal change in CLL [d]	Activity/ expr. in CLL [e]
NRF1(NRF)/MCF7-NRF1-ChIP-Seq(Unpublished)/Homer	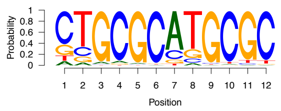	NRF1	Pro	ATAC loss<br>H3K4me3 ext.	down
NFY(CCAAT)/Promoter/ Homer	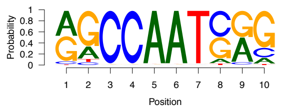	NFYA, NFYB	Pro	ATAC loss<br>H3K4me3 ext.<br>Nuc. gain	down/up
KLF3(Zf)/MEF-Klf3-ChIP-Seq(GSE44748)/Homer	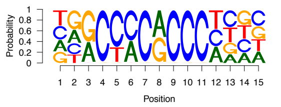	KLF3/11/16 [f]	Pro	ATAC loss<br>H3K4me3 ext.<br>Nuc. gain	down
Sp1(Zf)/Promoter/Homer	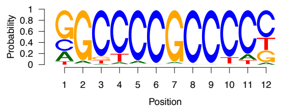	SP1	Pro	ATAC loss<br>H3K4me3 ext.<br>Nuc. gain	up
ERG(ETS)/VCaP-ERG-ChIP-Seq(GSE14097)/Homer	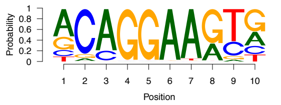	ETS2, ETV4, ELF4, SPI1 (PU.1), ERG	Pro<br>Enh	ATAC loss<br>H3K4me3 ext. (Pro)<br>DMR loss (Enh)<br>H3K27ac loss (Enh)<br>Nuc. gain (Enh)	down
Mef2c(MADS)/GM12878-Mef2c-ChIP-Seq(GSE32465)/Homer	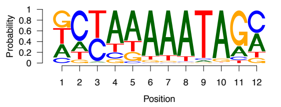	MEF2A, MEF2D	Pro<br>Enh	H3K4me3 loss (Pro)<br>ATAC loss (Enh)<br>DMR loss (Enh)<br>Nuc. gain (Enh)	down
E2A(bHLH)/proBcell-E2A-ChIP-Seq(GSE21978)/Homer	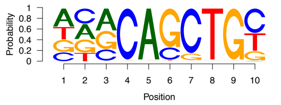	TCF4, TCF12 (E protein family)	Enh	ATAC gain<br>H3K27ac gain	up
TCFL2(HMG)/K562-TCF7L2-ChIP-Seq(GSE29196)/Homer	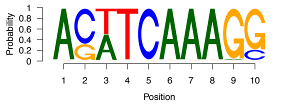	LEF1 (TCF7/LEF family)	Enh	ATAC gain<br>H3K27ac gain	up
NFAT(RHD)/Jurkat-NFATC1-ChIP-Seq(Jolma_et_al.)/Homer	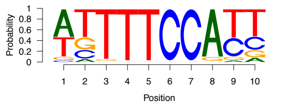	NFATC1	Enh	ATAC gain<br>DMR loss<br>H3K27ac gain	up
Foxo3(Forkhead)/U2OS-Foxo3-ChIP-Seq(E-MTAB-2701)/Homer	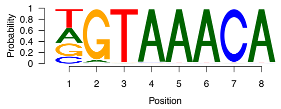	FOXP1, FOXN3 [g]	Enh	ATAC gain<br>DMR loss<br>H3K27ac gain	up
IRF8(IRF)/BMDM-IRF8-ChIP-Seq(GSE77884)/Homer	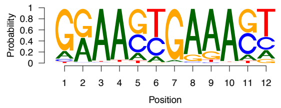	IRF4, IRF8	Enh	ATAC gain<br>DMR loss<br>H3K27ac gain	up
Egr1(Zf)/K562-Egr1-ChIP-Seq(GSE32465)/Homer	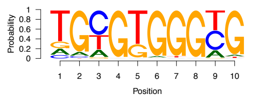	EGR1 [h]	Enh	ATAC gain<br>DMR loss<br>H3K27ac gain	down

Jun-AP1(bZIP)/K562-cJun-ChIP-Seq(GSE31477)/Homer	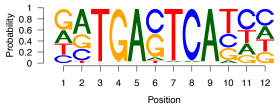	JUN, JUND, FOS, FOSL1/2 (AP-1)	Enh	ATAC loss H3K27ac loss	down
EBF(EBF)/proBcell-EBF-ChIP-Seq(GSE21978)/Homer	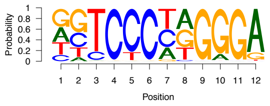	EBF1	Enh	ATAC loss H3K27ac loss	down, binding lost [i]
NFkB-p65(RHD)/GM12787-p65-ChIP-Seq(GSE19485)/Homer	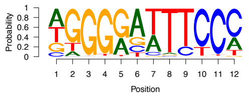	NFKB2, RELA, RELB (NF-κB)	Enh	ATAC loss H3K27ac loss	down
RUNX2(Runt)/PCa-RUNX2-ChIP-Seq(GSE33889)/Homer	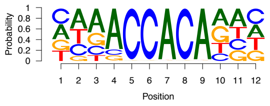	RUNX2/3	Enh	ATAC loss H3K27ac loss	down
CTCF(Zf)/CD4+-CTCF-ChIP-Seq(Barski_et_al.)/Homer	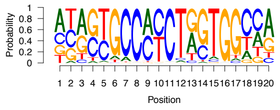	CTCF	Enh	ATAC loss H3K27ac loss	n. s., binding lost [i]

[a] The TF binding motifs and and sequence logos are from Homer and were identified from the analysis of regions with differential ATAC-seq signal that additionally were associated with the indicated changes of chromatin features in CLL.

[b] TF names are given according to the HUGO gene nomenclature committee (HGNC). Only those TFs were selected that showed a differential protein activity in CLL as opposed to other non-deregulated TFs that would recognize the same motif.

[c] Pro, promoters according to the RefSeq data base; Enh, predicted enhancers according to the chromatin context annotation but with the motif analysis being conducted only within ATAC-seq peak regions within the region that displayed the indicated differential signal (**Fig 5, S5**).

[d] H3K4me3 ext. refers to an extension of the H3K4me3 signal by 1-2 nucleosomes at promoters. H3K4me3 loss refers to promoters in a bivalent state (H3K4me3 and H3K27me3) that loose H3K4me3 in CLL. In addition, changes in the ATAC-seq signal, gain/loss of nucleosomes and differential DNA methylation and H3K27a acetylation at enhancers were considered (**Fig 6B**).

[e] In cases where the TF activity could not be reliably computed a change in gene expression (expr) was used if p-value < 0.01 and log fold change ≥ 1.7.

[f] KLF3/11/16 act as repressors via interactions with CTBP2 (KLF3) or with SINA3 (KLF11/16) and compete with SP1 for binding to similar sites (Kaczynski et al, 2003; McConnell & Yang, 2010).

[g] FOXP1 has 86 up- and 71 down-regulated target genes in the network. Thus, the protein appears to act both as an activator and repressor with an increased tendency to enhance gene expression. In addition to the canonical Forkhead motif GTAAACA, FOXN3 recognizes also the sequence GACGC (Rogers et al, 2019).

[h] The EGR1 binding motif was associated with a gain of enhancer activity in CLL although both its activity and gene expression were found to be downregulated. Thus, the protein might act as a repressor in CLL, which would be in line with previous reports on inhibitory activities of EGR1 (Feng et al, 2015; Thiel & Cibelli, 2002).

[i] The ChIP-seq analysis showed that EBF1 was lost at 826 sites and gained at 173 sites. Corresponding values for CTCF were 5964 and 441 sites in the absence of significant activity or expression changes.

Appendix Table S3. Comparison of EBF1 ChIP-seq data sets.

Sample type	Sample ID	EBF1 peaks, ENCODE	EBF1 peaks, this study	peak overlap (%)	Average overlap (%)	EBF1 expr. (RPKM)	Average expr. (RPKM) [b]
CLL IGHV-mut.	CLL6	356	4057	8.8		0.105	
CLL IGHV-mut.	CLL3	735	3411	21.5		0.050	
CLL IGHV-mut.	CLL13	8616	109483	7.9	16.4	0.052	0.20±0.12
CLL IGHV-mut.	CLL7	902	4809	18.8		0.675	
CLL IGHV-mut.	CLL4	4385	17362	25.3		0.095	
CLL IGHV-unmut.	CLL11	46	5600	0.8		0.045	
CLL IGHV-unmut.	CLL9	1487	4872	30.5	27.3	0.015	0.02±0.01
CLL IGHV-unmut.	CLL19	5335	10459	51.0		0	
CLL IGHV-unmut.	CLL08	1215	4544	26.7		0.025	
NBC	H14	768	3370	22.8		NA	
NBC	H15	594	3852	15.4	36.5	NA	48.25±3.82
NBC	H16	3113	6388	48.7		NA	
NBC	H17	6818	11583	58.9		NA	

[a] The EBF1 ChIP-seq data generated in this study were compared to peaks from the analysis of the ENCODE EBF1 ChIP-seq conducted with the human GM12878 cell line.

[b] Average and standard error of expression of the EBF1 gene in the different cell types. For NBC the average of samples H1, H3-5 and H7-9 was used. It is apparent that EBF1 is essentially silenced in both CLL IGHV mutated and unmutated samples as compared to the healthy references although there is a very low level of expression in IGHV mutated samples.

Appendix Table S4. Antibodies used in this study.

Antibodies	Source	Order number
Rabbit polyclonal H3	Abcam	ab1791
Rabbit polyclonal H3K4me1	Abcam	ab8895
Rabbit polyclonal H3K4me3	Abcam	ab8580
Rabbit polyclonal H3K9me3	Abcam	ab8898
Rabbit polyclonal H3K9ac	Active Motif	39917
Rabbit polyclonal H3K27ac	Abcam	ab4729
Rabbit polyclonal H3K36me3	Abcam	ab9050
Mouse monoclonal H3K27me3	Abcam	ab6002
Rabbit polyclonal beta-actin	Abcam	ab8227
Goat polyclonal EBF1	Sigma-Aldrich	SAB2501166
Rabbit polyclonal CTCF	Active Motif	61311

Appendix Table S5. ENCODE transcription factor binding site chromatin state enrichment.

**Cluster 1**	IKZF1, BCL11A, BATF
**Cluster 2**	EBF1, SPI1, MEF2C, NFATC1, IRF4, MEF2A
**Cluster 3**	RUNX3, NFIC, MTA3, FOXM1, ATF2
**Cluster 4**	ESR1, GATA2, ZNF217
**Cluster 5**	MAFK, MAFF, GATA3, FOS, FOXA2, CTCF, RAD21, FOXA1, POU5F1, EP300, STAT3, TAL1, CEBPB, JUN, FOSL2, RXRA, JUND, TEAD4, FOSL1, SMC3, HNF4G, HNF4A
**Cluster 6**	CTBP2, EZH2, SUZ12
**Cluster 7**	ZNF274, SETDB1
**Cluster 8**	KAP1, TRIM28, CBX3, FAM48A, BRF2, SIRT6, JUNB, GATA1, NR2F2, ARID3A, PRDM1, STAT5A, BCL3, PBX3, PAX5, POU2F2, WRNIP1, TCF3, TBL1XR1, RELA
**Cluster 9**	HDAC6, BACH1, CTCFL, YY1, ZZZ3, TCF7L2, REST, TFAP2A, TFAP2C, EGR1, MAZ, HDAC2, SMARCC1, MAX, MYC, NANOG, ZNF263, ZNF143, POLR2A, ESRRA, USF1, NR3C1, HDAC8, SMARCA4, RCOR1, TCF12, BHLHE40, SP1, USF2
**Cluster 10**	BRF1, POLR3G, KDM5A, GRp20, RDBP, THAP1, IRF3, SIX5, GTF2B, ELK4, ELK1, SAP30, PHF8, KDM5B, NRF1, SREBP1, SP2, ETS1, IRF1, NR2C2, NFYA, BRCA1
**Cluster 11**	ZBTB7A, FOXP2, E2F1, RBBP5, UBTF, E2F6, SIN3A, SMARCB1, GTF2F1, HSF1, ZBTB33, TAF1, ZKSCAN1, GABPA, CHD1, CEBPD, CCNT2, SIN3AK20, HMGN3, SP4, CREB1, HDAC1, TAF7, E2F4, BDP1, RPC155, SMARCC2, STAT1, ATF3, SRF, NFYB, GTF3C2, RFX5, MYBL2, PPARGC1A, STAT2, ATF1, NFE2, MBD4, PML, BCLAF1, ZEB1, MXI1, TBP, ELF1, CHD2

Analysis of the ENCODE transcription factor binding sites was performed using ChromHMM overlap enrichment using default parameters. For the enrichment of chromatin states of these 161 transcription factors, we identified 11 clusters, which were averaged and are shown in **Fig. EV4B**. Selection of the clusters was determined based on the dendogram of hierarchical clustering of the patterns using the hclust function in R.

Appendix Table S6. Inventory of appendix data sets.

File Name	Figure/ table ref.	Description
Dataset_EV01-overview_datasets.xlsx	Fig 1-6, EV1-5, S1-5	Overview of the appendix data sets EV2-14.
Dataset_EV02-samples.xlsx	Fig 1, Fig S1, Table S1	Sample meta data describing sample names, origin, sex, alternative IDs and taxonomic data.
Dataset_EV03-meC.xlsx<br>3.1 CLL-PMDs	Fig 2, Fig EV2, Fig S2	Merged consensus list of PMDs identified. The consensus PMDs were generated as the union of PMDs present in at least half of the CLL samples analyzed (≥6). Data are in BED format.
Dataset_EV03-meC.xlsx<br>3.2 DMRs	Fig 2, Fig EV2, Fig S2	DMRs identified comparing CLL to NBC samples. The R tools DSS was used with default parameters, with p-value < 0.05 in regions at least 200 bp with a minimum methylation change of 0.3. Data are in BED format.
Dataset_EV04-histone-ChIPseq-QC.xlsx	Fig 1	Quality parameters describing histone ChIPseq quality for each sample, and inclusion of sample in ChromHMM model generation and differential histone modification analysis.
Dataset_EV05-promoters.xlsx<br>5.1 H3K4me3 broad	Fig 3, Fig EV3	Promoters with H3K4me3 broadening in CLL. Promoters must exhibit a significant (p-value < 0.05) gain of at least 2 nucleosomes (400 bp). Data are in BED format.
Dataset_EV05-promoters.xlsx<br>5.2 H3K4me3 broad and nuc. gain	Fig 3, Fig EV3	Promoters (TSS +/- 1 kb) which gain nucleosomes in CLL. Data are in BED format.
Dataset_EV05-promoters.xlsx<br>5.3/5.4 Bivalent promoters lost/gained	Fig 3, Fig EV3	Bivalent promoters as identified by the clustering in Fig 3F. Data are in BED format.
Dataset_EV06-ChromHMM-model.xlsx<br>6.1-6.4 ChromHMM model	Fig 1	Chromatin 12 state model emission parameters (6.1), state descriptions (6.2), transition parameters (6.3), model (6.4). 6.1, 6.3-6.4 are directly from the output of ChromHMM model generation.
Dataset_EV06-ChromHMM-model.xlsx<br>6.5 Links to 12 state model	Fig 1	Hyperlinks to the 12 state chromatin segmentations of samples including all CLL, NBC, and panobinostat/mock treated samples at 24 hours. Linked Data are in BED format.
Dataset_EV07-ChromHMM-merged-states.xlsx	Fig 1	Merged chromatin states occuring in at least 3 samples (A-L) for each chromatin state. Data are in BED format.
Dataset_EV08-ChIPseq-dif_histone-mod_CTCF_EBF1.xlsx<br>8.1 Histone modifications	Fig 3-6	Differential histone modifications regions for each of the 7 histone modifications comparing CLL to NBCs using only samples passing QC. Data are in BED format.
Dataset_EV08-ChIPseq-dif_histone-mod_CTCF_EBF1.xlsx<br>8.2 Diff H3K27ac PS 24 h	Fig 4, Fig S4	Differential H3K27ac at enhancer regions between panobinostat treated and control CLL samples at 24 hours (using CLL + PS 24h enhancers as background). Analysis performed using DiffBind paired analysis with a FDR threshold of 0.01.
Dataset_EV08-ChIPseq-dif_histone-mod_CTCF_EBF1.xlsx	Fig 5	Differential ChIP-seq of CTCF comparing CLL to NBCs. Data are in BED format.

8.3 CTCF ChIP-seq		
Dataset_EV08-ChIPseq-dif_histone-mod_CTCF_EBF1.xlsx<br>8.4 EBF1 ChIP-seq	Fig 6	Differential ChIP-seq of EBF1 comparing CLL to NBCs. Data are in BED format.
Dataset_EV09-enhancers.xlsx<br>9.1-9.6 CLL and NBC	Fig 4,<br>Fig EV4,<br>Fig S4	Putative enhancer regions from ChromHMM states 1, 8, 9, 11 and super-enhancer called from H3K27ac and ATAC in CLL + NBC, CLL, NBC. Data are in BED format.
Dataset_EV09-enhancers.xlsx<br>9.7-9.9 Differential enhancers	Fig 4,<br>Fig EV4,<br>Fig S4,<br>Fig 5	Differential predicted (super-)enhancers loci between CLL and NBC samples (min3) as well as enhancer state changes that occur in panobinostat treated samples. Analysis performed using DiffBind with FDR threshold of 0.01. Data are in BED format.
Dataset_EV09-enhancers.xlsx<br>9.10-9.11 Active enhancers	Fig 4,<br>Fig 5,<br>Fig S6,<br>Fig 6,<br>Fig S8	“Active enhancers in CLL” (occuring in at least 3 patients) or “active enhancers in NBC” (occuring in at least 1 NBC) determined from ATAC-seq.
Dataset_EV09-enhancers.xlsx<br>9.12-9.13 Active Bidi enhancers	Fig EV4,<br>Fig S4	Consensus list of active enhancers with bidirectional transcription in CLL (occuring in at least 3 patients) or NBC (occuring in at least 2 NBC).
Dataset_EV10-ATAC.xlsx<br>10.1 Differential ATAC	Fig 1,<br>Fig EV1,<br>Fig 3,<br>Fig 5,<br>Fig S8	Differential ATAC peaks between CLL and NBC (fixed and viably frozen samples). Analysis was performed using DiffBind with thresholds of FDR 0.00095 and fold change < -2.15 or fold change > 1.55. Data are in BED format.
Dataset_EV10-ATAC.xlsx<br>10.2 Diff ATAC (only fixed samples)	Fig S5	Differential ATAC peaks between all fixed processed CLL and NBC samples. Analysis was performed using DiffBind with thresholds of FDR 0.00759 and fold change < -1.5 or fold change > 1.8. Data are in BED format.
Dataset_EV10-ATAC.xlsx<br>10.3 HOMER-Gained diff ATAC enh	Fig 5	Homer analysis results of enriched motifs in gained ATAC sites between CLL and NBC within potential enhancer regions regions defined by ChromHMM states 1, 8, 9 and 11.
Dataset_EV10-ATAC.xlsx<br>10.4 HOMER-Lost diff ATAC enh	Fig 5	Homer analysis results of enriched motifs in lost ATAC sites between CLL and NBC within potential enhancer regions defined by ChromHMM states 1, 8, 9 and 11.
Dataset_EV10-ATAC.xlsx<br>10.5 Used known HOMER motifs	Fig 5	Position weight matrices of known motifs used for HOMER motif analysis.
Dataset_EV10-ATAC.xlsx<br>10.6/10.7scATAC-seqPE-pairs	Fig 5,<br>Fig S6	Enhancer-promoter pairs that showed the highest correlations in CLL and NBCs within a 200 kb window.
Dataset_EV10-ATAC.xlsx<br>10.8-10.10 Differential CTCF sites	Fig 5,<br>Fig EV5,<br>Fig S6	Differential ATAC peaks overlapping with CTCF binding sites in the GM12878 lymphoblast cell line (ENCODE). Data are in BED format.
Dataset_EV10-ATAC.xlsx<br>10.11 cons. ATAC peaks	Fig 5,<br>Fig S5	ATAC-seq peak regions found in at least four replicates across all CLL samples and NBC controls (consensus region list derived with DiffBind).

Dataset_EV11-RNAseq-dif-gene-expr.xlsx<br>11.1 Differential expression of CLL genes	Fig 1,<br>Fig 6,<br>Fig S7	Gene expression differences in CLL vs NBC identified with DEseq2 (gencode 17).
Dataset_EV11-RNAseq-dif-gene-expr.xlsx<br>11.2 Diff intronic RNA PS 24h	Fig 4,<br>Fig S4	Gene expression differences of nascent transcripts. Analysis was performed using DESeq2 on intronic RNA reads as a proxy for nascent transcript levels between panobinostat treated and control CLL samples at 24 hours.
Dataset_EV12-chromatin-expr-changes.xlsx	Fig 6	Co-occurence of differential gene expression and changes of chromatin features in CLL.
Dataset_EV13-Bcell-network.xlsx<br>13.1 ARACNE network	Fig 1,<br>Fig 6,<br>Fig S7	Gene regulatory B cell network constructed with ARACNE-AP (default parameters) based on the B cell gene expression data set from Basso et al (Basso et al, 2005). The text file includes all the regulator to target gene interactions, the type of interaction and its likelihood. The network can be visualized with Cytoscape.
Dataset_EV13-Bcell-network.xlsx<br>13.2 VIPER	Fig 1,<br>Fig 6,<br>Fig S7	Regulator activities calculated with VIPER using the regulatory B cell network and the RNA-seq data from our study. The table shows all regulators and differential activity between CLL and control samples.
Dataset_EV13-Bcell-network.xlsx<br>13.3 Enhancer-promoter interact	Fig 1,<br>Fig 6,<br>Fig S7	Enhancer-promoter interactions with a correlation of 0.22 or higher from the scATAC-seq analysis that were integrated into the network.
Dataset_EV13-Bcell-network.xlsx<br>13.4 CLL-specific GREN	Fig 1,<br>Fig 6,<br>Fig S7	Gene regulatory enhancer containing network that includes the connected part of the transcription factors, chromatin modifiers and target genes deregulated in CLL from our study.
Dataset_EV13-Bcell-network.xlsx<br>13.5 Aberrant factors CLL	Fig 1,<br>Fig 6,<br>Fig S7	Overview of all deregulated genes from the different analyses.
Dataset_EV14_CLLspecific_GREN.cys	Fig 1,<br>Fig 6,<br>Fig S7	Gene regulatory enhancer containing network that includes the connected part of the transcription factors, chromatin modifiers and target genes deregulated in CLL from our study for visualization with the Cytoscape viewer. For each TF in Table S2 a subnetwork was created.

Appendix Table S7. Data analysis software.

Software	Ref.	Link
Bowtie	(Langmead et al, 2009)	bowtie-bio.sourceforge.net/bowtie2/index.shtml
MACS	(Zhang et al, 2008)	https://github.com/taoliu/MACS
SICER	(Zang et al, 2009)	github.com/razZ0r/sicer
ChromHMM	(Ernst & Kellis, 2012)	compbio.mit.edu/ChromHMM/
MCORE	(Molitor et al, 2017).	malone.bioquant.uni-heidelberg.de/software/mcore/
NucTools	(Vainshtein et al, 2017)	generegulation.info/index.php/nuctools
HOMER	(Heinz et al, 2010)	homer.ucsd.edu/homer/
ROSE	(Loven et al, 2013; Whyte et al, 2013)	bitbucket.org/young_computation/rose
GREAT	(McLean et al, 2010)	bejerano.stanford.edu/great/public/html/index.php
STAR	(Dobin et al, 2013)	github.com/alexdobin/STAR
DiffBind	(Ross-Innes et al, 2012)	doi.org/10.18129/B9.bioc.DiffBind
chromVAR	(Schep et al, 2017)	greenleaflab.github.io/chromVAR/
ARACNe-AP	(Lachmann et al, 2016)	sourceforge.net/projects/aracne-ap/
VIPER	(Alvarez et al, 2016)	doi.org/10.18129/B9.bioc.viper
Cytoscape	(Shannon et al, 2003)	cytoscape.org
DAVID	(Huang da et al, 2009)	david.ncifcrf.gov
gProfileR	(Reimand et al, 2016)	https://biit.cs.ut.ee/gprofiler/
DESeq2	(Love et al, 2014)	doi.org/10.18129/B9.bioc.DESeq2
GenomicRanges	(Lawrence et al, 2013)	doi.org/10.18129/B9.bioc.GenomicRanges
Trimmomatic	(Bolger et al, 2014)	http://www.usadellab.org/cms/?page=trimmomatic
Bioconductor R packages: GenomicRanges, Gviz, DSS, minfi, EnrichedHeatmap,	(Gentleman et al, 2004)	www.bioconductor.org
BWA	(Li & Durbin, 2010)	bio-bwa.sourceforge.net
methylCtools	(Hovestadt et al, 2014)	https://github.com/hovestadt/methylCtools
Integrative Genomics Viewer	(Robinson et al, 2011)	software.broadinstitute.org/software/igv/
4D Genome data base	(Teng et al, 2014)	https://4dgenome.research.chop.edu/Download.html
SAMtools	(Li et al, 2009)	http://samtools.sourceforge.net/
bedtools	(Quinlan & Hall, 2010)	http://bedtools.readthedocs.io/en/latest/
Centipede	(Pique-Regi et al, 2011)	http://centipede.uchicago.edu
Custom analysis scripts	This paper	https://github.com/CancerEpiSys/Mallm-et-al-processing-scripts
RWire	This paper	https://github.com/FabianErdel/RWire

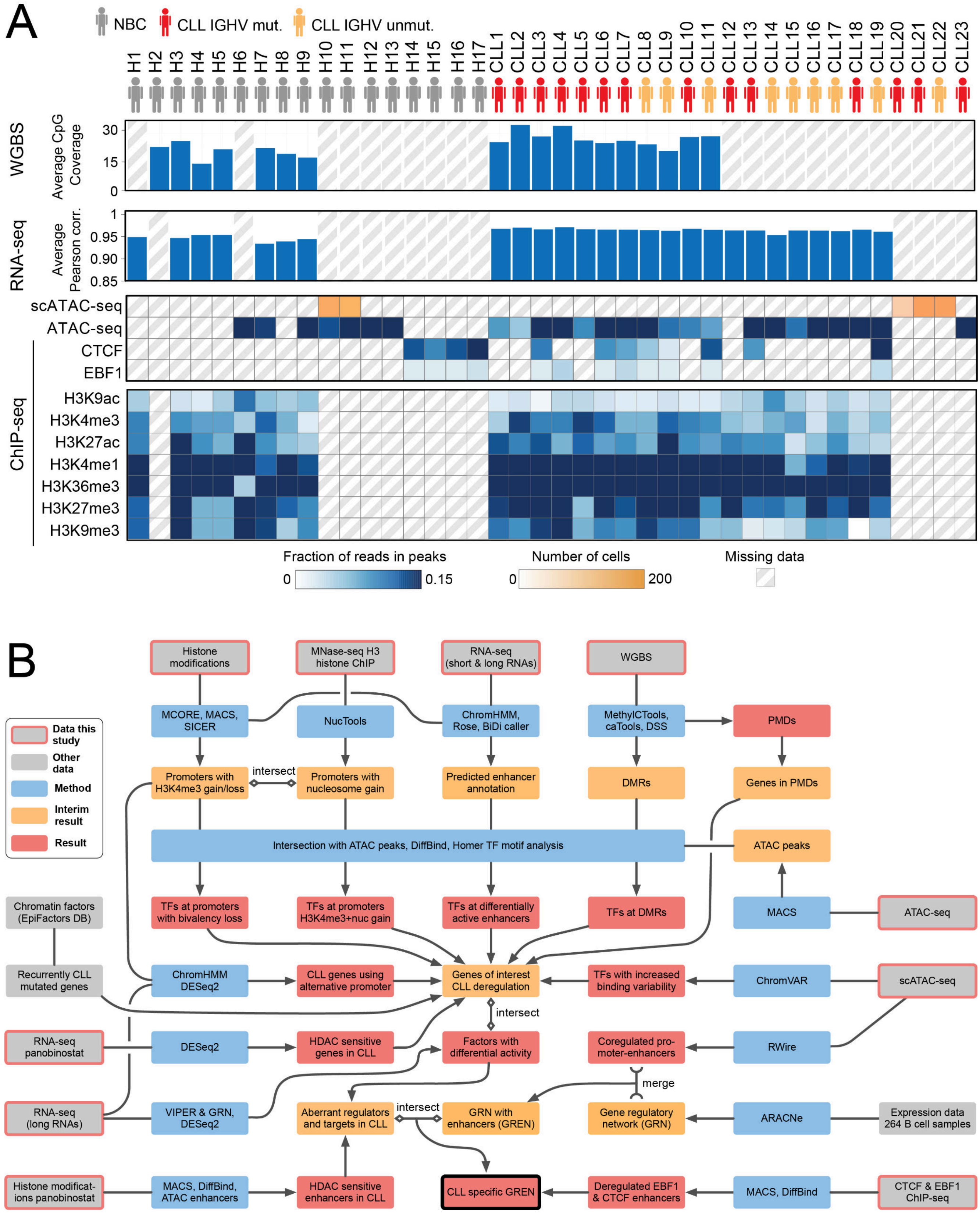

Appendix Figure S1. Data quality assessment and analysis workflow.

(**A**) The top panel lists all the CLL samples derived from individual CLL patients and NBC controls collected from healthy donors. Bottom panels show the quality metrics of genome-wide chromatin

feature and gene expression profiles. Data sets missing for individual samples are marked with striped bars. Average coverage at CpG sites was evaluated for the WGBS data, and low-coverage sample H4 was removed from further DNA methylation analysis. Variation in sample preparation for RNA-seq was assessed using average Pearson correlation among NBC and CLL samples. All samples displayed Pearson correlations >0.94 and were included in the analysis. The fraction of reads in peaks and relative strand cross-correlation were used to assess the quality of ATAC-seq and ChIP-seq samples as visualized in the heatmap. Only high-quality ChIP-seq data sets were included for the ChromHMM model generation step. For scATAC-seq cells with a very high or very low number of integrations were removed (Materials and Methods). **(B)** Data analysis workflow. The data generated in this study (grey, red outline) together with data from the literature (grey) were analyzed with the indicated methods (blue, **Appendix Table S7**). From the intersection of deregulated chromatin features with ATAC-seq peaks a set of TFs was identified based on a binding motif analysis. Those TFs that also displayed a differential activity were selected to extract the connected part of the B cell regulatory network (GRN, **Dataset EV13**). From the scATAC-seq analysis, promoter and enhancers that showed high correlations for being simultaneously accessible in the same cell were extracted. This enhancer-promoter network was integrated with the GRN to create a gene regulatory enhancer containing network (GREN, **Dataset EV14**). From the GREN a “CLL specific GREN” was extracted. It connected the TFs, their target genes as well as linked chromatin modifiers affecting the aberrant chromatin features that were identified. Additionally, it was required that selected factors were deregulated in their activity and/or expression between CLL and NBCs.

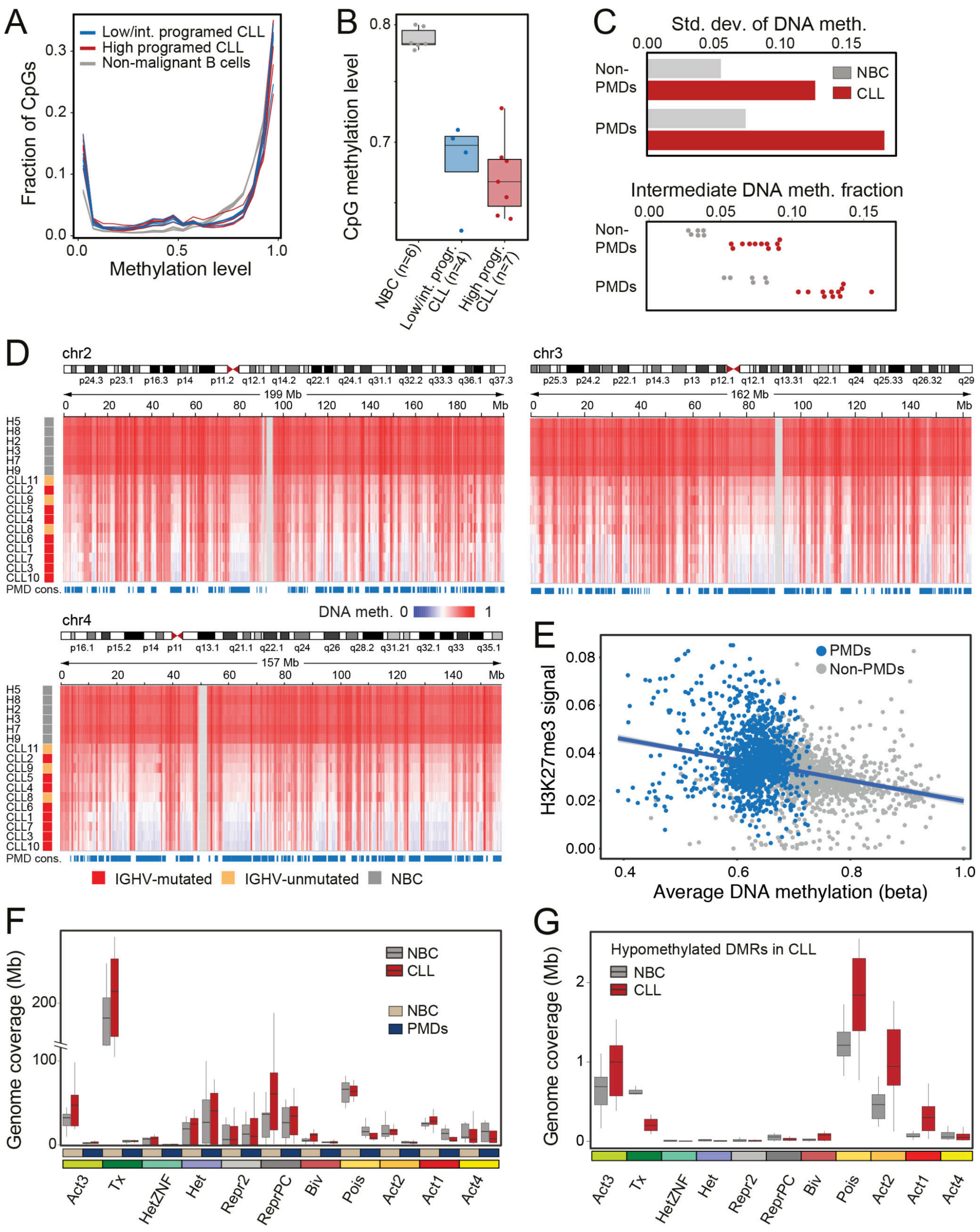

Appendix Figure S2. Characterization of CLL-specific DNA methylation patterns.

(A) Distribution of DNA methylation levels in CLL and NBC samples. **(B)** Genome-wide CpG methylation categorized into three groups: NBC (grey), low/intermediate programmed CLL (blue) and high programmed CLL (red). **(C)** Top: Average standard deviation of DNA methylation across samples.

Bottom: Fraction of intermediate DNA methylation (β-values between 0.4 and 0.6). **(D)** Examples of large partially methylated domains (PMDs) on chromosome 2, 3, and 4 derived from a consensus of CLL samples (*n*=11) in comparison to the NBC reference (*n*=6). **(E)** Scatter plot of H3K27me3 signal with DNA methyation in PMDs (blue) and non-PMD (grey) regions in CLL. H3K27me3 and meC were anticorrelated and on average PMDs had a higher H3K27me3/meC ratio, which points to a more silenced chromatin state. **(F)** Genome coverage of ChromHMM chromatin states as defined in **Fig 1B** for CLL (red) and NBC (grey) overlapping with PMD and non-PMD regions. **(G)** Distribution of chromatin states in differentially methylated regions (DMRs). DMRs were specifically enriched for the putative active (Act2, Act3) or poised (Pois) enhancer states.

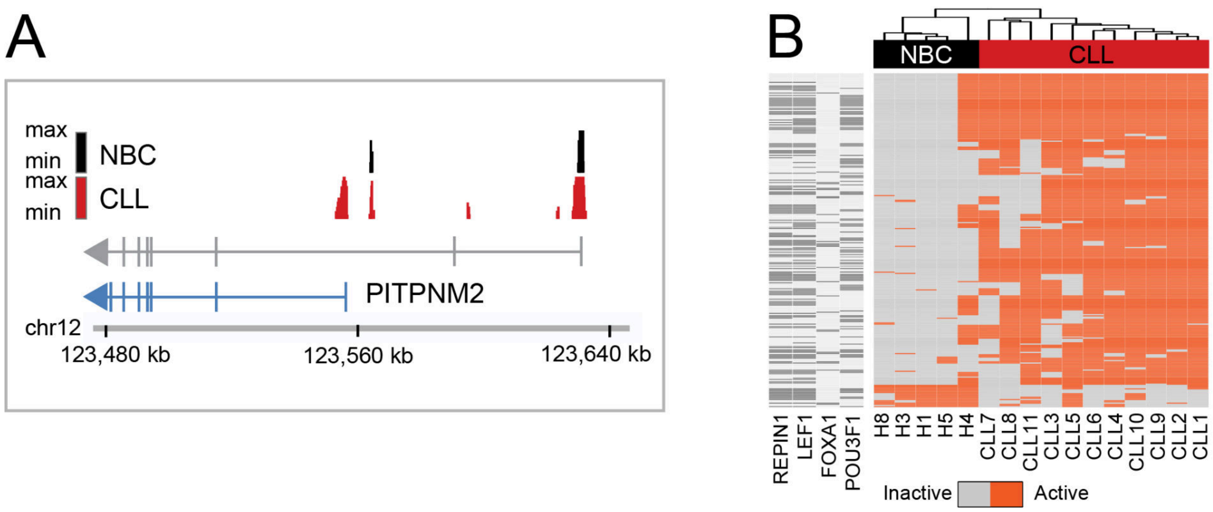

Appendix Figure S3. Alternative promoter usage in CLL cells.

(A) Exemplary region for an alternative promoter used in CLL that leads to an alternative transcript of the PITPNM2 gene. The signal shown corresponds to the active TSS state from ChromHMM analysis. The height of signal represents the number of samples in the active TSS state, and the maximum was set to all available samples for NBC controls and CLL patients. Gene tracks show transcriptome annotation estimated by StringTie. Blue color indicates upregulation in CLL compared to NBCs (FDR<0.05) and grey no differential expression detected (FDR>0.05). **(B)** Heatmap representing the activity of alternative promoters defined by ChromHMM annotation. Orange represents active promoters, grey inactive promoters overlapping with known Gencode transcripts. Only recurrent alternative promoters are shown. The left columns indicate in dark grey transcripts with a significant correlation between REPIN1, LEF1, FOXA1 and POU3F1 expression and alternative promoter usage ($p < 0.05$), while light grey indicates a lack of correlation ($p > 0.05$).

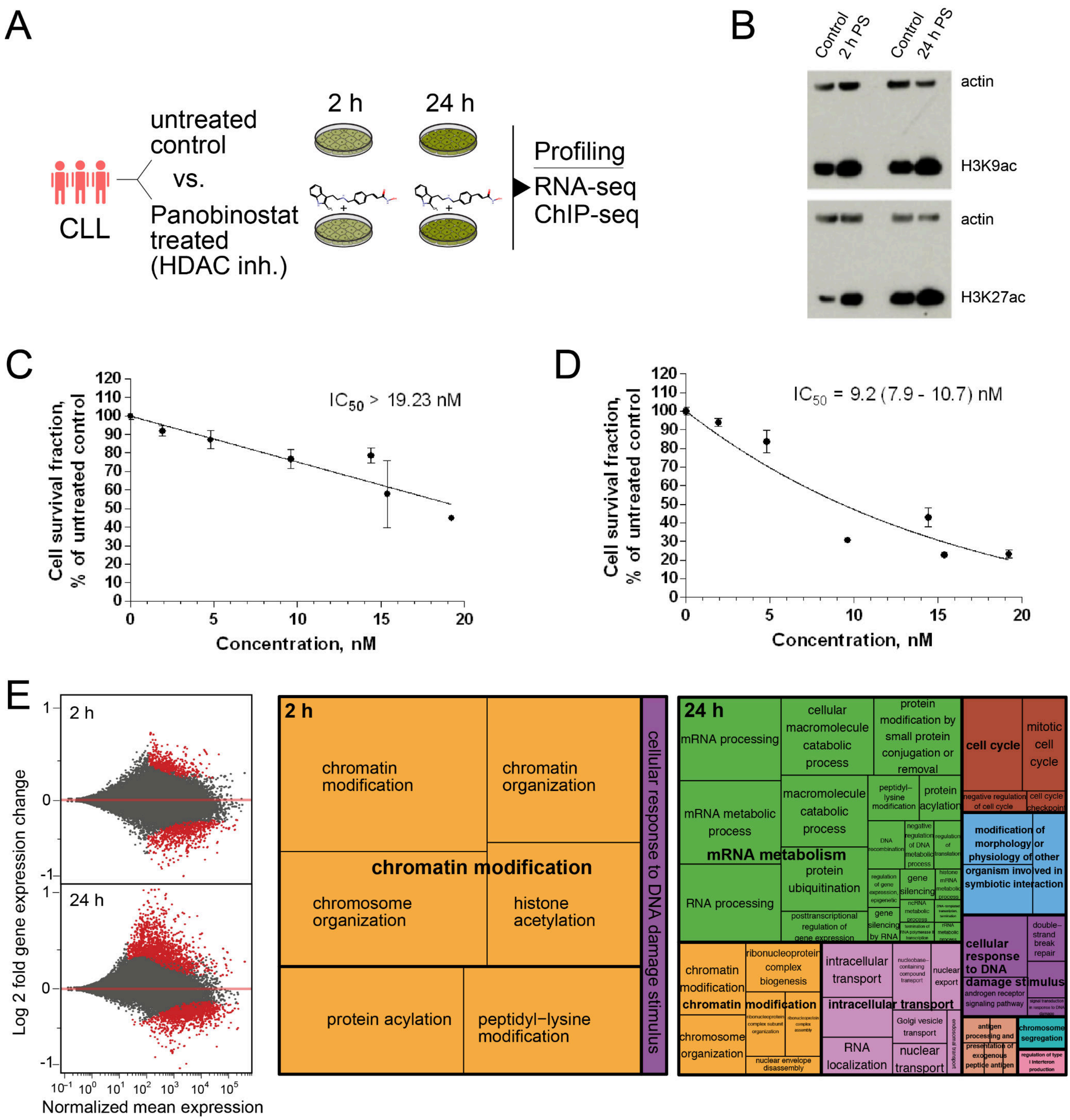

Appendix Figure S4. Response of CLL cells to HDAC inhibition.

(**A**) Scheme of ex-vivo treatment with the HDAC inhibitor panobinostat for 2 h or 24 h followed by RNA-seq and ChIP-seq ananlysis. **(B)** Western blot of cell lysates from CLL cells that were treated with 5 nM panobinostat (PS) for 2 or 24 hours. Control cells were mock-treated with DMSO and cultured under the same conditions as panobinostat-treated cells. Detection with antibodies specifically recognizing acetylated lysine 9 (H3K9ac) or lysine 27 (H3K27ac) of histone 3 show a significant enrichment of acetylation levels in panobinostat-treated samples compared to mock-treated controls. β-actin served as loading control. **(C)** Survival of primary B cells upon incubation with panobinostat. After 24 hours of ex-vivo treatment with panobinostat at different concentrations, survival of primary CD19+ sorted B cells of a healthy donor was assessed by quantification of apoptotic cells with annexin and 7-AAD in flow cytometry. **(D)** Survival of CLL cells after incubation with panobinostat. Same as in panel C but for

CD19+ sorted B cells from a CLL patient. **(E)** Gene expression changes determined by intronic read counts using DESeq2. Log2 fold change above 0 indicates higher expression after treatment. Transcripts with BH q-value < 0.01 were considered to be differentially expressed. The response was more pronounced after 24 h (bottom plot) with up to 3-fold change in expression. Biological processes with enrichment of genes downregulated upon panobinostat treatment were identified with GREAT pathway analysis. Enriched GO terms were semantically clustered to superordinate terms. While affected genes at 2 h are primarily overrepresented in chromatin modification and histone acetylation, several additional processes are enriched after 24 h. Larger square sizes indicate smaller BH q-values of corresponding GO terms.

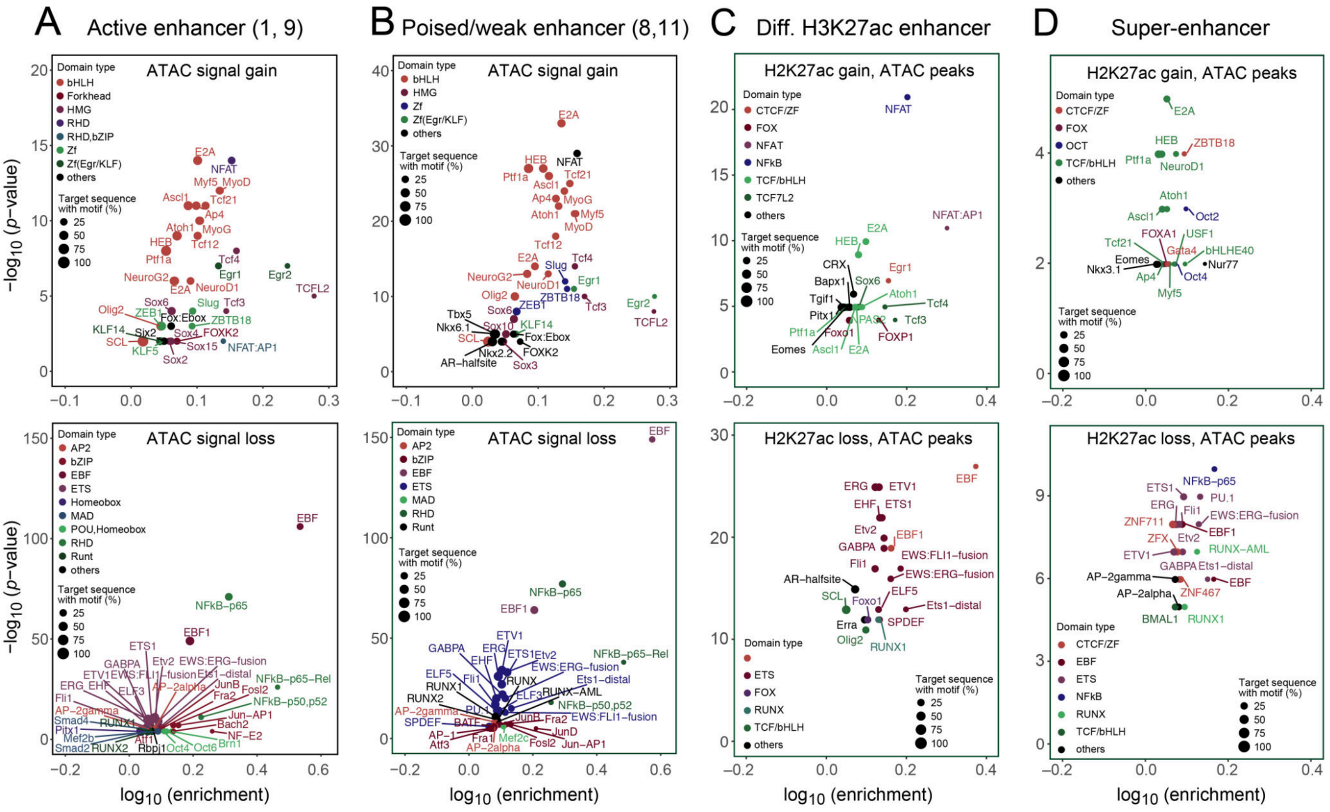

Appendix Figure S5. TF binding motif analysis at enhancers predicted by additional annotations.

(A) Plot of the most enriched TF binding motifs in regions that showed gained (top) or lost (bottom) ATAC-seq signal (Appendix Dataset_EV10.2) at predicted enhancer regions represented by ChromHMM states 1 and 9. Similar TF binding motifs have the same color and represent binding motif classes. The size of the spots is proportional to the percentage of target sequences with that motif. The -log10 *p*-values (y-axis) were plotted against the log10 enrichment (x-axis) of sites with the motif in enhancers that gained/lost the ATAC signal using all ATAC-seq peak regions in the indicated ChromHMM states as background model. **(B)** Same as panel A but for poised/weak predicted enhancers regions from ChromHMM states 8 and 11. **(C)** Same as panel A but for ATAC peak regions at predicted enhancers annotated from H3K27ac peaks that gained (top) or lost (bottom) H3K27ac. **(D)** Same as in panel C but for super-enhancers called with the Rose software tool as described in Materials and Methods.

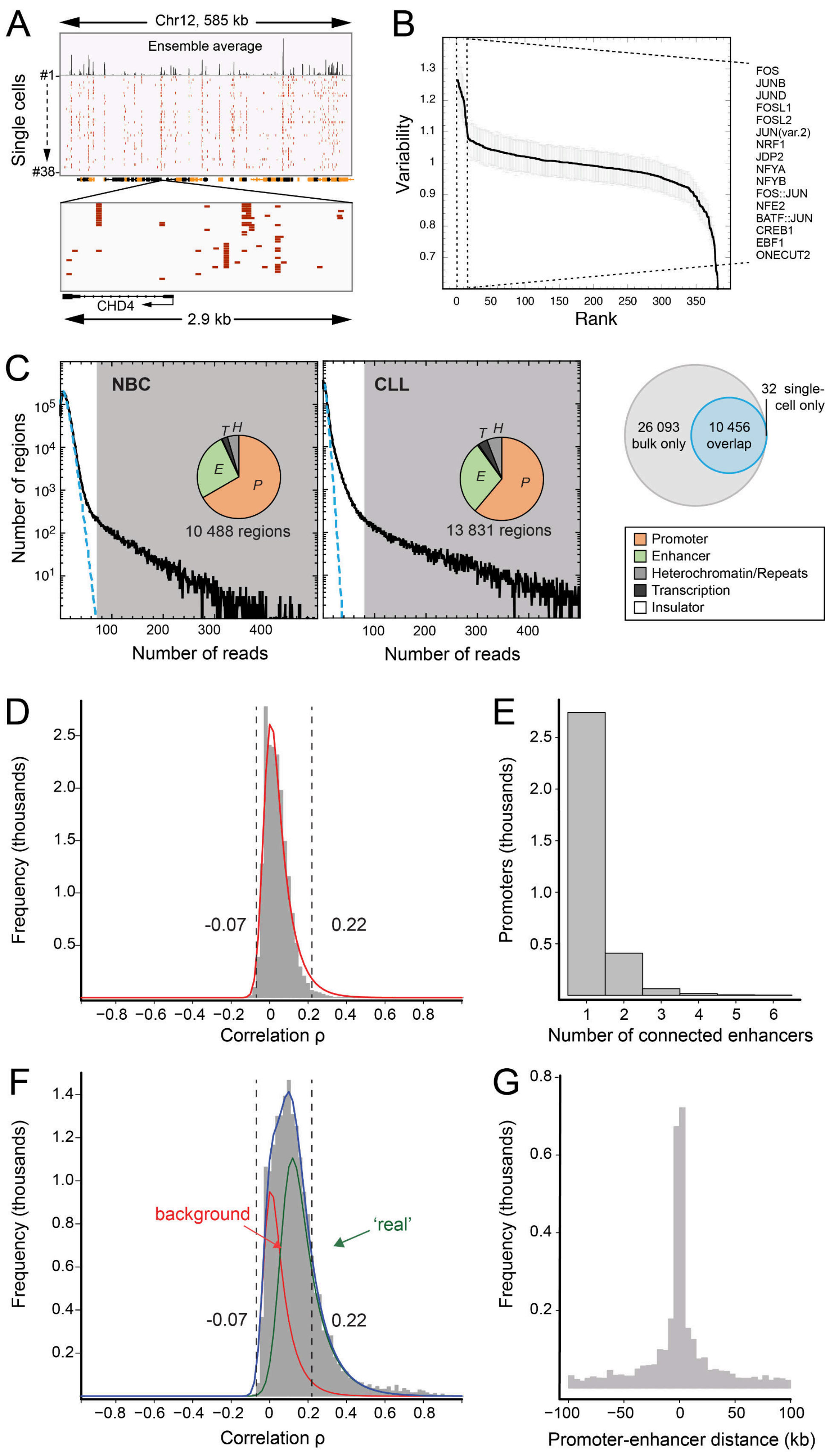

Appendix Figure S6. Single cell ATAC-seq analysis.

(**A**) Single cell analysis of chromatin accessibility by ATAC-seq (scATAC-seq) from a total of 343 NBCs (2 donors) and 494 CLL cells (3 patients). Some ATAC insertion sites displayed a strong co-occurrence

in a subset of single cells. (**B**) Variation in accessibility of different TF binding motifs across CLL and NBCs. To the right, the 16 TF binding motifs that displayed the highest variability for being present in the open or closed state are listed. Most of the motifs were also found in the analysis of aberrant CLL chromatin features (**Appendix Table S2**). (**C**) The number of reads observed for each genomic region (1 kb tiles) was plotted. Blue dashed lines represent non-negative binomial distributions to approximate the background population while gray areas highlight open regions. With the given threshold (70 reads in 343 NBCs, see gray area in panel A) essentially all open regions identified by scATAC-seq were also present in the bulk ATAC-seq data from the same sample. **(D)** Estimation of correlation 'background' signal. For each promoter, the integration numbers were permuted across cells. Distribution values: mean, 0.028; median, 0.019; variance, 0.004; 1% threshold, ρ = -0.074; 99% threshold, ρ = 0.220. Red line: Exponentially modified Gaussian distribution. (**E**) Number of enhancers per promoter for correlations with ρ > 0.22. (**F**) Distribution of promoter-enhancer pair correlations. Distribution values: mean, 0.028; median, 0.019; variance, 0.004. Lines: Exponentially modified Gaussian distributions. (**G**) Distribution of promoter-enhancer distances for correlation ρ > 0.22. For promoters with multiple enhancers, only the highest-correlated one was considered. Of these promoter-enhancer pairs 72 % (CLL) and 74 % (NBC) derived from our scATAC-seq analysis were listed also in the 4D Genome data base (https://4dgenome.research.chop.edu/Download.html) as interactions for Homo sapiens. When considering only 4D genome interactions within the 10%-90% distance range of all 4D genome interactions (7.23 kb to 429 kb), these numbers changed to 58 % (CLL) and 56 % (NBCs).

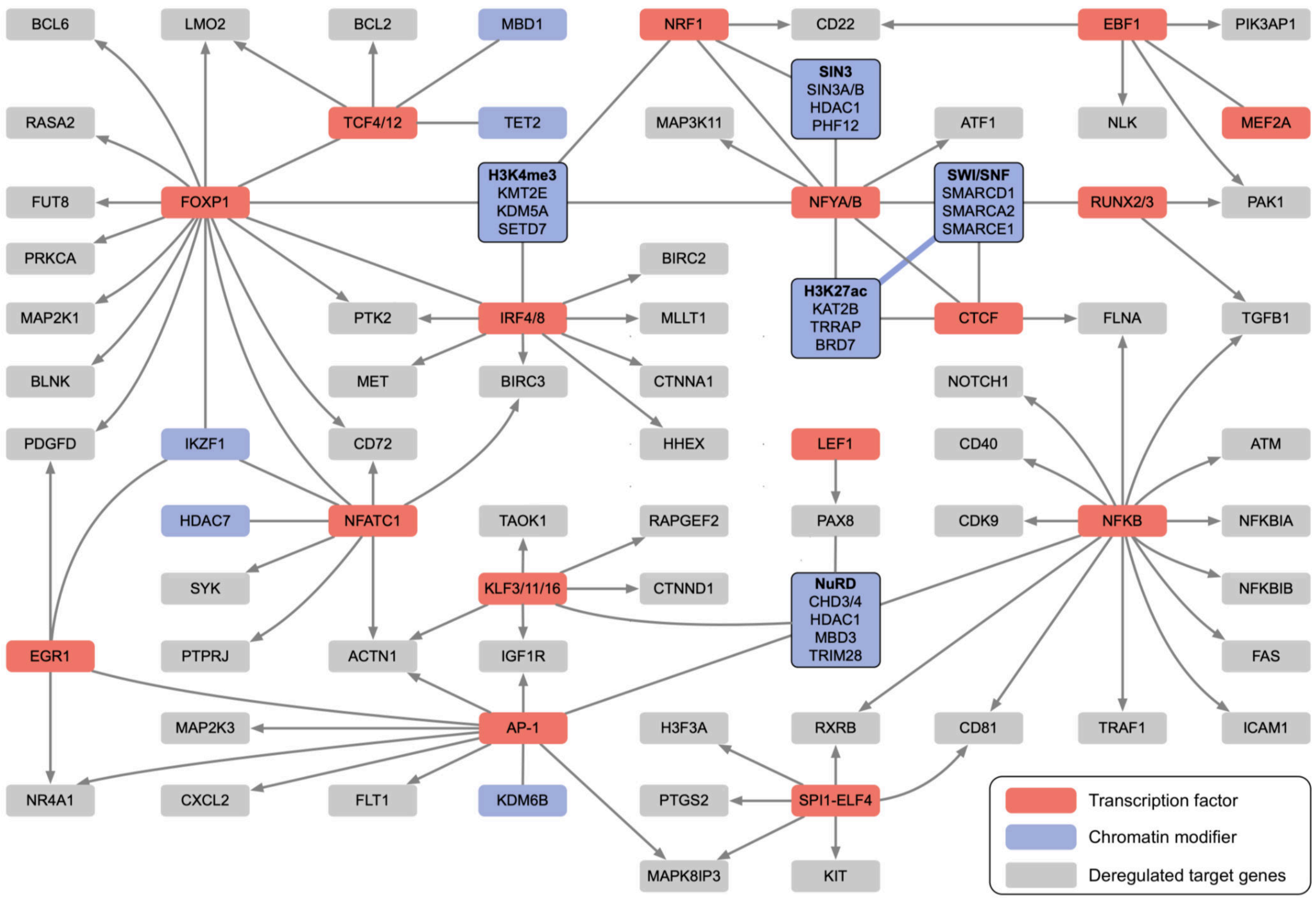

Appendix Figure S7. CLL TF network with linked deregulated chromatin modifiers and targets.

A simplified TF network for CLL with associated chromatin modifiers and TF target genes from the enriched pathways is depicted (see also **Fig 6C** and **Appendix Table S2**). It was extracted from the complete CLL GREN (**Dataset EV14**) and included only factors with deregulated activity/expression between CLL and NBCs. Chromatin modifiers were selected by intersecting the genes deregulated in CLL with the Epifactors database (Medvedeva et al, 2015). From this set, genes were chosen that had activities linked to the deregulated chromatin features identified in our study (meC loss, H3K27ac loss/gain and H3K4me3 loss/gain, see **Fig 6B**) and were linked to the TFs identified in the core network. These chromatin modifiers were grouped according to histone modification type and association into known complexes. The latter includes the two main repressive HDAC containing complexes SIN3 and NuRD (Ahringer, 2000) that have been associated with crucial functions in oncogenesis and cancer progression (Lai & Wade, 2011). Furthermore, we find a number of deregulated subunits of the chromatin remodeling complex SWI/SNF (BAF) in our network. The 15 subunits of this complex are frequently found to be mutated in human cancers and play an important role in transcriptional activation (Kadoch & Crabtree, 2015). It is noted that loss of SWI/SNF activity leads to a loss of H3K27ac and enhancer activity via interaction of SWI/SNF with p300 (Alver et al, 2017; Hodges et al, 2018). This connection between SWI/SNF and H3K27ac at enhancers is indicated in the network by a blue line. IKZF1 protein is linked to FOXP1, EGR1 and NFATC1 and has been reported to target both the NuRD and SWI/SNF complexes to chromatin (O'Neill et al, 2000). Finally, a recent reports shows that enhancer binding of basic helix-loop-helix TFs recognizing the E box site was dependent on TET2 (Rasmussen et al, 2019), which showed an increased activity in CLL and displayed a link to TCF12 in our network.

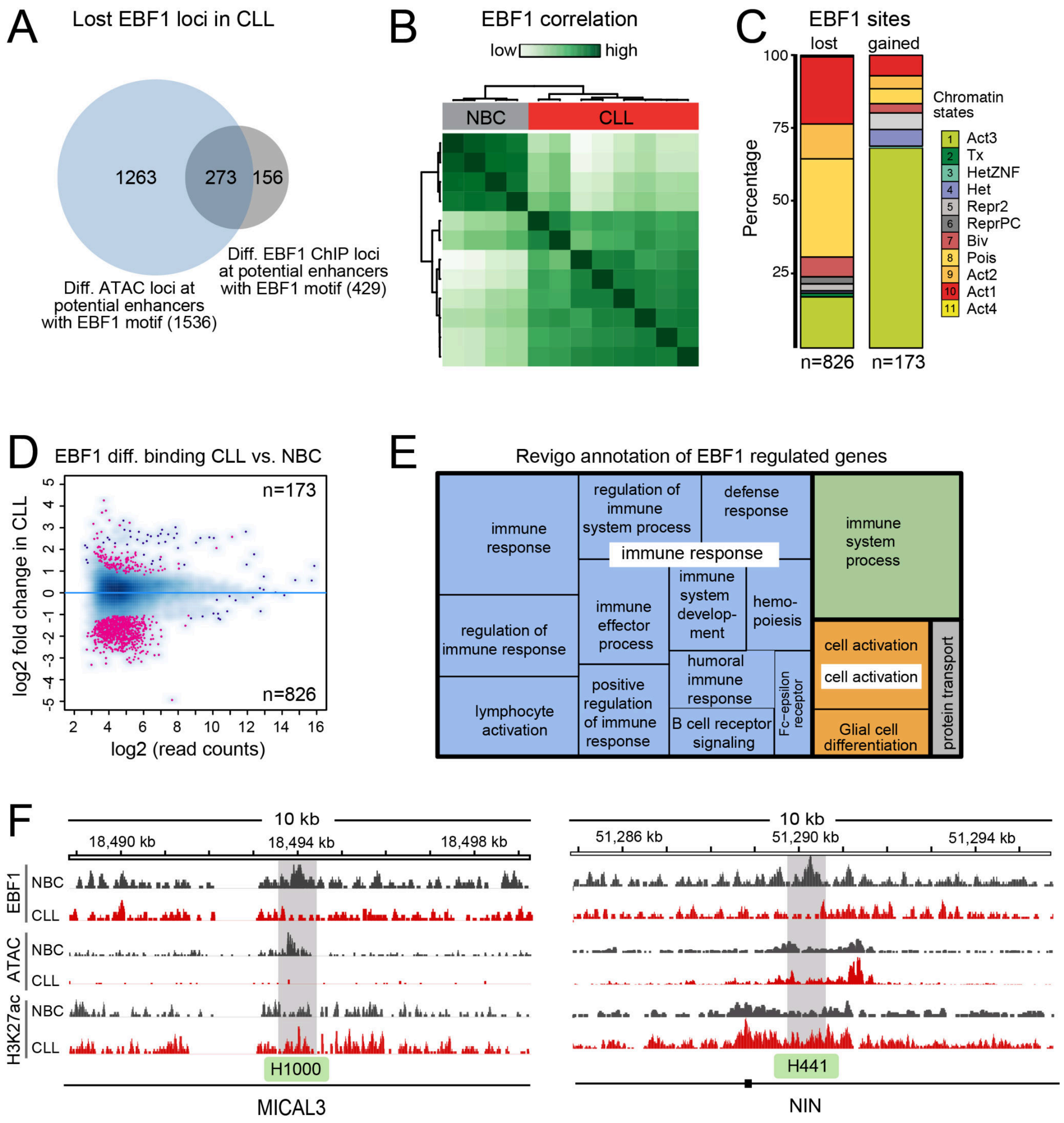

Appendix Figure S8. Analysis of EBF1 binding by ChIP-seq.

(A) Overlap of regions that lost EBF1 ChIP-seq and ATAC-seq signal in CLL at enhancers containing the EBF1 motif. **(B)** Unsupervised clustering of EBF1 ChIP-seq read occupancy. EBF1 was detected at 5358 loci in CLL cells and 6298 sites in NBCs. CLL and NBC samples were clearly separated based on the EBF1 ChIP-seq signal. **(C)** ChromHMM annotation of EBF1 peaks lost in CLL (*n* = 840) and gained in CLL (*n* = 173). Loss of EBF1 predominantly occurred at the transcription start site (Act1 state) and at putatively poised enhancers (Pois state), while EBF1 binding was gained mainly at active, genic sites (Act 3 state). (**D**) MA-plot of differentially occupied EBF1 ChIP-seq regions. A large scale loss of EBF1 binding activity in CLL was observed. **(E)** Annotation of EBF1 regulated genes. The enrichment

of GO terms of lost EBF1 binding regions at enhancers points to a role of EBF1 in immune response, immune system process, cell activation and regulation of protein transport. (**F**) Analysis of EBF1 binding to predicted enhancers of its target genes *MICAL3* and *NIN*. Both genes displayed a loss of EBF1 binding at their enhancers H1000 and H441 as predicted from our CLL GREN. Similar to H464 of *SNX22* (**Fig 6E**) these enhancers were active in NBCs but inactive in CLL. For enhancer H401 of *NIN*, no differential EBF1 binding was detected (data not shown). Thus, 3 out of 4 of EBF1 target genes with silenced enhancers in CLL displayed a loss of EBF1 binding in the ChIP-seq analysis, which was predicted from our network.

Appendix References

Ahringer J (2000) NuRD and SIN3 histone deacetylase complexes in development. Trends Genet 16: 351-356

Alvarez MJ, Shen Y, Giorgi FM, Lachmann A, Ding BB, Ye BH, Califano A (2016) Functional characterization of somatic mutations in cancer using network-based inference of protein activity. Nat Genet 48: 838-847

Alver BH, Kim KH, Lu P, Wang X, Manchester HE, Wang W, Haswell JR, Park PJ, Roberts CW (2017) The SWI/SNF chromatin remodelling complex is required for maintenance of lineage specific enhancers. Nat Commun 8: 14648

Basso K, Margolin AA, Stolovitzky G, Klein U, Dalla-Favera R, Califano A (2005) Reverse engineering of regulatory networks in human B cells. Nat Genet 37: 382-390

Bolger AM, Lohse M, Usadel B (2014) Trimmomatic: a flexible trimmer for Illumina sequence data. Bioinformatics 30: 2114-2120

Dobin A, Davis CA, Schlesinger F, Drenkow J, Zaleski C, Jha S, Batut P, Chaisson M, Gingeras TR (2013) STAR: ultrafast universal RNA-seq aligner. Bioinformatics 29: 15-21

Ernst J, Kellis M (2012) ChromHMM: automating chromatin-state discovery and characterization. Nat Methods 9: 215-216

Feng Y, Desjardins CA, Cooper O, Kontor A, Nocco SE, Naya FJ (2015) EGR1 Functions as a Potent Repressor of MEF2 Transcriptional Activity. PLoS ONE 10: e0127641

Gentleman RC, Carey VJ, Bates DM, Bolstad B, Dettling M, Dudoit S, Ellis B, Gautier L, Ge Y, Gentry J, Hornik K, Hothorn T, Huber W, Iacus S, Irizarry R, Leisch F, Li C, Maechler M, Rossini AJ, Sawitzki G et al (2004) Bioconductor: open software development for computational biology and bioinformatics. Genome Biol 5: R80

Heinz S, Benner C, Spann N, Bertolino E, Lin YC, Laslo P, Cheng JX, Murre C, Singh H, Glass CK (2010) Simple combinations of lineage-determining transcription factors prime cis-regulatory elements required for macrophage and B cell identities. Mol Cell 38: 576-589

Hodges HC, Stanton BZ, Cermakova K, Chang CY, Miller EL, Kirkland JG, Ku WL, Veverka V, Zhao K, Crabtree GR (2018) Dominant-negative SMARCA4 mutants alter the accessibility landscape of tissue-unrestricted enhancers. Nat Struct Mol Biol 25: 61-72

Hovestadt V, Jones DTW, Picelli S, Wang W, Kool M, Northcott PA, Sultan M, Stachurski K, Ryzhova M, Warnatz H-J, Ralser M, Brun S, Bunt J, Jäger N, Kleinheinz K, Erkek S, Weber UD, Bartholomae CC, von Kalle C, Lawerenz C et al (2014) Decoding the regulatory landscape of medulloblastoma using DNA methylation sequencing. Nature 510: 537-541

Huang da W, Sherman BT, Lempicki RA (2009) Systematic and integrative analysis of large gene lists using DAVID bioinformatics resources. Nat Protoc 4: 44-57

Kaczynski J, Cook T, Urrutia R (2003) Sp1- and Kruppel-like transcription factors. Genome Biol 4: 206

Kadoch C, Crabtree GR (2015) Mammalian SWI/SNF chromatin remodeling complexes and cancer: Mechanistic insights gained from human genomics. Sci Adv 1: e1500447

Lachmann A, Giorgi FM, Lopez G, Califano A (2016) ARACNe-AP: gene network reverse engineering through adaptive partitioning inference of mutual information. Bioinformatics 32: 2233-2235

Lai AY, Wade PA (2011) Cancer biology and NuRD: a multifaceted chromatin remodelling complex. Nat Rev Cancer 11: 588-596

Langmead B, Trapnell C, Pop M, Salzberg SL (2009) Ultrafast and memory-efficient alignment of short DNA sequences to the human genome. Genome Biol 10: R25

Lawrence M, Huber W, Pages H, Aboyoun P, Carlson M, Gentleman R, Morgan MT, Carey VJ (2013) Software for computing and annotating genomic ranges. PLoS Comput Biol 9: e1003118

Li H, Durbin R (2010) Fast and accurate long-read alignment with Burrows-Wheeler transform. Bioinformatics 26: 589-595

Li H, Handsaker B, Wysoker A, Fennell T, Ruan J, Homer N, Marth G, Abecasis G, Durbin R, Genome Project Data Processing S (2009) The Sequence Alignment/Map format and SAMtools. Bioinformatics 25: 2078-2079

Love MI, Huber W, Anders S (2014) Moderated estimation of fold change and dispersion for RNA-seq data with DESeq2. Genome Biol 15: 550

Loven J, Hoke HA, Lin CY, Lau A, Orlando DA, Vakoc CR, Bradner JE, Lee TI, Young RA (2013) Selective inhibition of tumor oncogenes by disruption of super-enhancers. Cell 153: 320-334

McConnell BB, Yang VW (2010) Mammalian Kruppel-like factors in health and diseases. Physiol Rev 90: 1337-1381

McLean CY, Bristor D, Hiller M, Clarke SL, Schaar BT, Lowe CB, Wenger AM, Bejerano G (2010) GREAT improves functional interpretation of cis-regulatory regions. Nat Biotechnol 28: 495-501

Medvedeva YA, Lennartsson A, Ehsani R, Kulakovskiy IV, Vorontsov IE, Panahandeh P, Khimulya G, Kasukawa T, Consortium F, Drablos F (2015) EpiFactors: a comprehensive database of human epigenetic factors and complexes. Database (Oxford) 2015: bav067

Molitor J, Mallm JP, Rippe K, Erdel F (2017) Retrieving Chromatin Patterns from Deep Sequencing Data Using Correlation Functions. Biophys J 112: 473-490

O'Neill DW, Schoetz SS, Lopez RA, Castle M, Rabinowitz L, Shor E, Krawchuk D, Goll MG, Renz M, Seelig HP, Han S, Seong RH, Park SD, Agalioti T, Munshi N, Thanos D, Erdjument-Bromage H, Tempst P, Bank A (2000) An ikaros-containing chromatin-remodeling complex in adult-type erythroid cells. Mol Cell Biol 20: 7572-7582

Pique-Regi R, Degner JF, Pai AA, Gaffney DJ, Gilad Y, Pritchard JK (2011) Accurate inference of transcription factor binding from DNA sequence and chromatin accessibility data. Genome Res 21: 447-455

Quinlan AR, Hall IM (2010) BEDTools: a flexible suite of utilities for comparing genomic features. Bioinformatics 26: 841-842

Rasmussen KD, Berest I, Kebetaler S, Nishimura K, Simon-Carrasco L, Vassiliou GS, Pedersen MT, Christensen J, Zaugg JB, Helin K (2019) TET2 binding to enhancers facilitates transcription factor recruitment in hematopoietic cells. Genome Res 29: 564-575

Reimand J, Arak T, Adler P, Kolberg L, Reisberg S, Peterson H, Vilo J (2016) g:Profiler-a web server for functional interpretation of gene lists (2016 update). Nucleic Acids Res 44: W83-89

Robinson JT, Thorvaldsdottir H, Winckler W, Guttman M, Lander ES, Getz G, Mesirov JP (2011) Integrative genomics viewer. Nat Biotechnol 29: 24-26

Rogers JM, Waters CT, Seegar TCM, Jarrett SM, Hallworth AN, Blacklow SC, Bulyk ML (2019) Bispecific Forkhead Transcription Factor FoxN3 Recognizes Two Distinct Motifs with Different DNA Shapes. Mol Cell 74: 245-253 e246

Ross-Innes CS, Stark R, Teschendorff AE, Holmes KA, Ali HR, Dunning MJ, Brown GD, Gojis O, Ellis IO, Green AR, Ali S, Chin SF, Palmieri C, Caldas C, Carroll JS (2012) Differential oestrogen receptor binding is associated with clinical outcome in breast cancer. Nature 481: 389-393

Schep AN, Wu B, Buenrostro JD, Greenleaf WJ (2017) chromVAR: inferring transcription-factor-associated accessibility from single-cell epigenomic data. Nat Methods 14: 975-978

Shannon P, Markiel A, Ozier O, Baliga NS, Wang JT, Ramage D, Amin N, Schwikowski B, Ideker T (2003) Cytoscape: a software environment for integrated models of biomolecular interaction networks. Genome Res 13: 2498-2504

Thiel G, Cibelli G (2002) Regulation of life and death by the zinc finger transcription factor Egr-1. J Cell Physiol 193: 287-292

Vainshtein Y, Rippe K, Teif VB (2017) NucTools: analysis of chromatin feature occupancy profiles from high-throughput sequencing data. BMC Genomics 18: 158

Whyte WA, Orlando DA, Hnisz D, Abraham BJ, Lin CY, Kagey MH, Rahl PB, Lee TI, Young RA (2013) Master transcription factors and mediator establish super-enhancers at key cell identity genes. Cell 153: 307-319

Zang C, Schones DE, Zeng C, Cui K, Zhao K, Peng W (2009) A clustering approach for identification of enriched domains from histone modification ChIP-Seq data. Bioinformatics 25: 1952-1958

Zhang Y, Liu T, Meyer CA, Eeckhoute J, Johnson DS, Bernstein BE, Nusbaum C, Myers RM, Brown M, Li W, Liu XS (2008) Model-based analysis of ChIP-Seq (MACS). Genome Biol 9: R137
