# Supplementary material for: Linking aberrant chromatin features in chronic lymphocytic leukemia to transcription factor networks
Source: Mol Syst Biol. 2019 May 22;15(5):e8339. doi: 10.15252/msb.20188339 (PMC6529931; doi:10.15252/msb.20188339)
Supplement: Supplementary file 2 — Expanded View Figures PDF [file MSB-15-e8339-s002.pdf]

Expanded View Figures

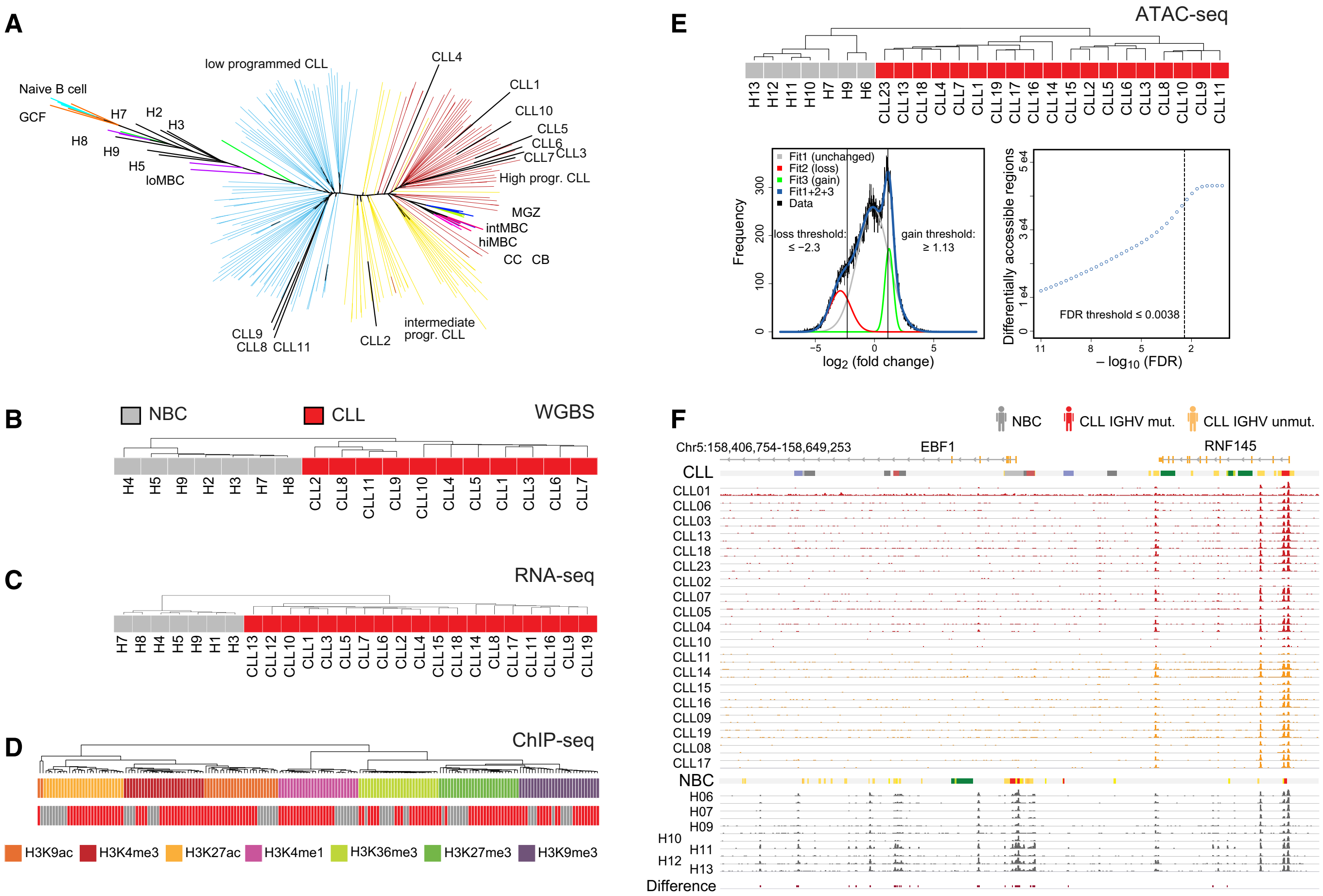

Figure EV1. Data set overview.

A Assignment of CLL samples to B-cell developmental stages based on DNA methylation patterns.

B Unsupervised hierarchical clustering of the samples from Pearson's correlation coefficient (average linkage) for DNA methylation from WGBS. The analysis was carried out considering the most variable 1 million CpG sites.

C Same as panel (B) but computed from the gene expression profiles of 2,000 genes from RNA-seq.

D Same as panel (B) but computed for histone modifications at promoters from ChIP-seq. Samples cluster according to the modifications, underlining specificity of the experimental data, and separate inactive (H3K9me3 and H3K27me3) from the other active histone marks.

E Top: Unsupervised hierarchical clustering of the samples from Spearman's correlation coefficient (average linkage) for chromatin accessibility from ATAC-seq calculated from ~ 120,000 accessible regions. Bottom: Distribution of fold changes in ATAC-seq signal from DiffBind between CLL and NBC samples. The data were fitted to a sum of three Gaussian functions. Threshold values were determined from the indicated cross-over points as described in Materials and Methods.

F Exemplary comparison of ATAC-seq data of all analyzed CLL IGHV mutated (n = 11), CLL IGHV unmutated (n = 8), and NBC (n = 7) samples in replicates (except for H10, H12, and H13) at the *EBF1* locus. It contains regions with lost ATAC-seq signal in CLL as compared to NBC controls determined by the DiffBind analysis (red bars in bottom track "Difference"). Representative ChromHMM state annotations of CLL1 and H6 are depicted as color bars above the corresponding group.

© 2019 The Authors

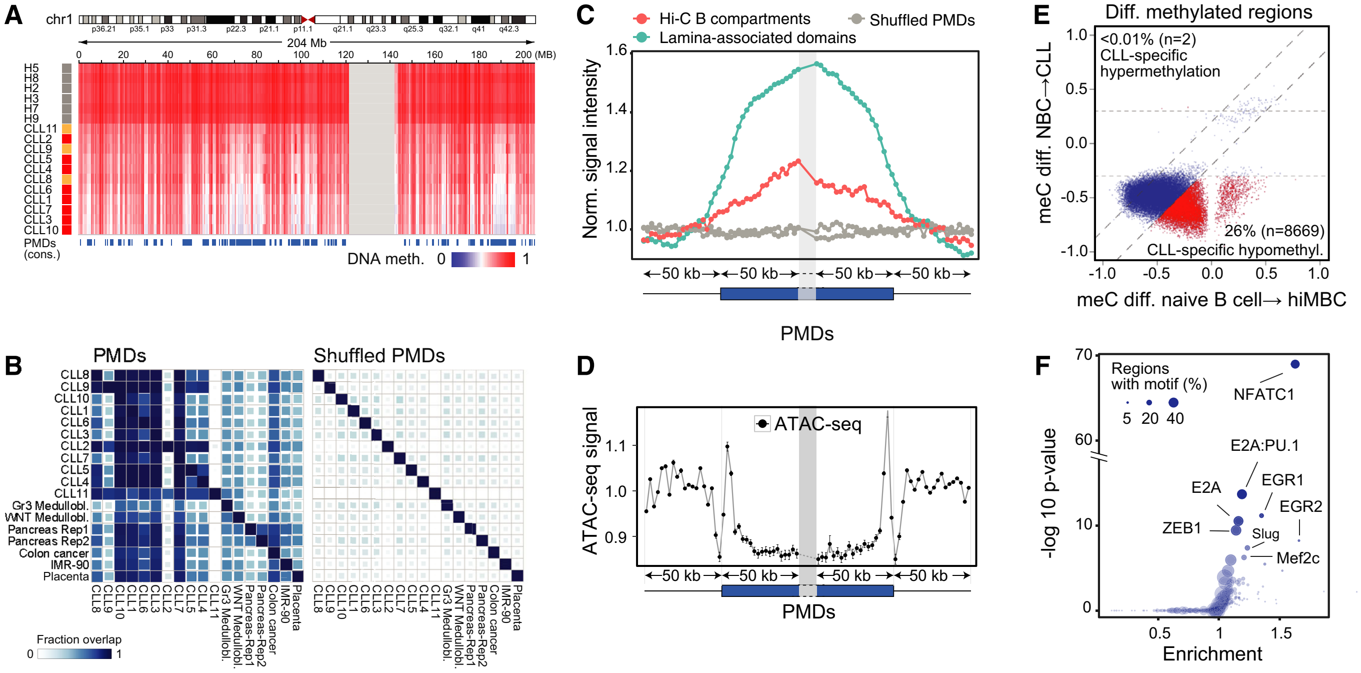

Figure EV2. Characterization of partially methylated domains and DMRs.

A Example of a large PMD on chromosome 1 derived from a consensus of CLL samples (n = 11) in comparison with the NBC reference (n = 6).

B Similarity of PMDs found in CLL, medulloblastoma (Group3/WNT; Hovestadt *et al*, 2014), colon cancer (Berman *et al*, 2011), pancreas (Schultz *et al*, 2015), and placenta tissues (Schroeder *et al*, 2013). Medullobl., medulloblastoma; Gr3, group3; Rep, replicate. Lower triangular part refers to the fraction of the PMD on the y-axis overlapping with the PMD sample on the x-axis and vice versa for the upper triangular.

C Normalized coverage plot of lamina-associated domains (Guelen *et al*, 2008) and Hi-C B compartments (Fortin & Hansen, 2015) around the ± 50 kb flanking regions of PMD boundaries in 2.5-kb windows.

D Distribution of ATAC around the ± 50 kb flanking regions of PMD boundaries in 5-kb windows. Normalized fold changes were calculated by dividing to the average signal flanking outside the PMD boundaries. Blue box, within PMDs; thin line, outside PMDs, nor.—normalized.

E Methylation changes in DMRs from CLL vs. NBCs plotted against the methylation changes of the same regions during normal B-cell maturation from naive B cells to class-switched memory B cells (hiMBC). Data for DMRs were averaged across multiple CpG sites and across replicate samples for each experimental class. CLL-specific DMRs (red, CLL-specific) displayed a higher change of more than 0.2 b-value in comparison with B-cell programming. The parameter n indicates the total number of CLL-specific DMRs, hypo- and hyper-methylated.

F Enrichment of TF binding motifs at ATAC-seq peaks overlapping with DMRs. As background, all the consensus ATAC-seq peaks outside the DMRs were used. Size indicates the percentage of DMR-overlapping ATAC-seq peaks with the motif.

© 2019 The Authors

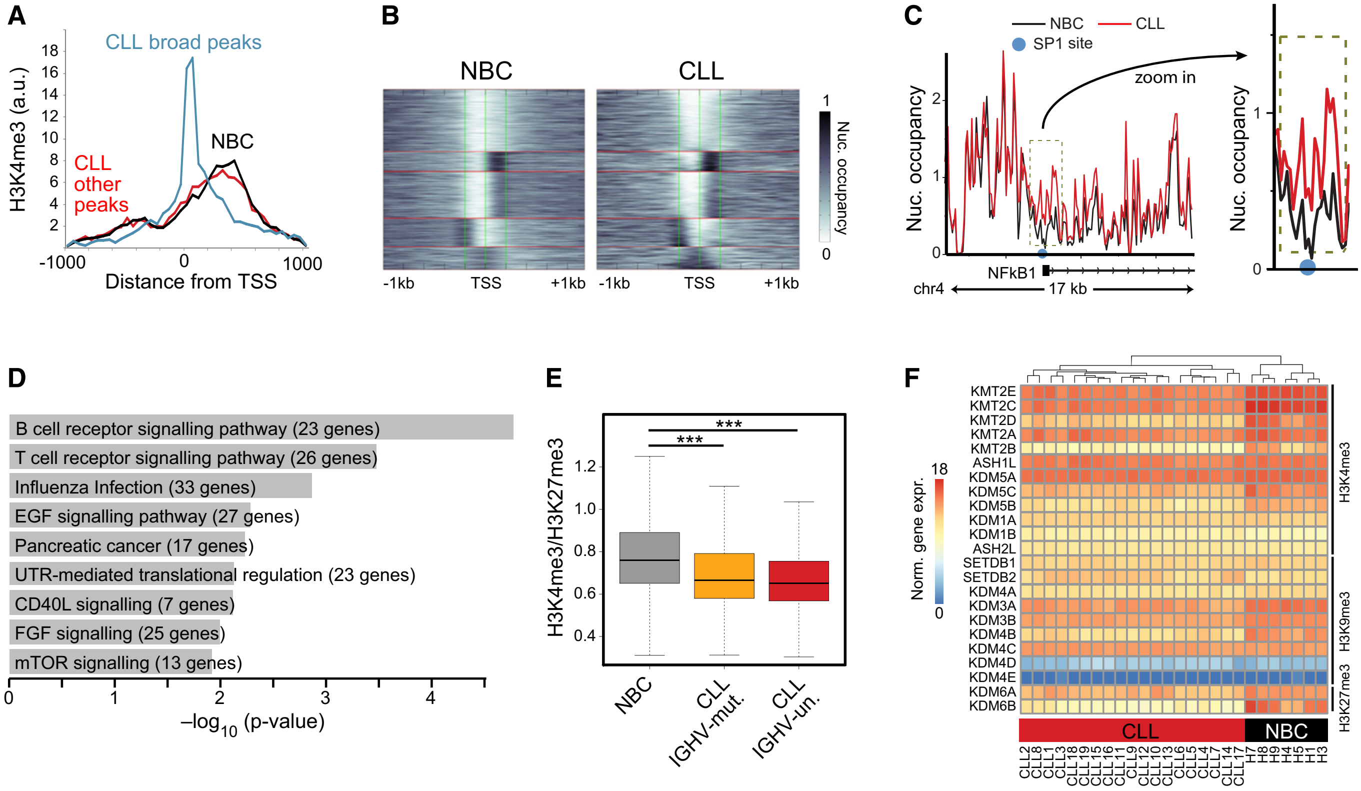

Figure EV3. Analysis of H3K4me3 peak broadening and loss of bivalent states at CLL promoters.

A H3K4me3 distribution around TSS for NBCs and CLL. Both samples types displayed a similar H3K4me3 distribution up- and downstream of the TSS. However, for CLL extended peaks were found that centered around the TSS, indicating a gain of nucleosomes in these regions.

B Cluster plot of nucleosomes occupancy at all CLL-specific promoters that gain nucleosomes in CLL. Left: K-means clustering of nucleosome occupancy for NBC samples. Right: CLL samples with the same ordering as for NBC controls. A fraction of promoters in the bottom cluster displayed a particularly pronounced gain of nucleosomes at the TSS, which reflects the profile of the promoters with the extended H3K4me3 signal depicted in panel (A).

C Nucleosome profile at the promoter region of *NFKB1* with higher nucleosome density for CLL at the TSS (red line) compared to NBCs (black line). The light blue circle indicates SP1 binding sites in the lymphoblastoid cell line GM12878.

D GO enrichment analysis of genes with gained nucleosomes at their promoters.

E Ratio of read counts for H3K4me3 and H3K27me3 at bivalent promoters for CLL samples grouped according to their IGVH mutation status and compared to NBC samples (***$P < 0.001$, t-test).

F Cluster heatmap of histone demethylase and methyltransferase expression values. Samples were clustered according to expression similarities (hierarchical clustering), while enzymes were sorted according to their target modification.

Figure EV4. Comparison of enhancers identified with previous data sets and exemplary loci.

A Overlap of CLL and NBC annotated active chromatin states (1, 8, 9, and 11) identified here with previous data sets. Venn diagram showing the total chromatin (Mb) of recurrent predicted enhancer chromatin state, occurring in at least three samples, compared to published corresponding states E7–11 of peripheral blood $CD19^+$ B cells and $CD3^+$ T cells from the Roadmap Epigenome project. In our union set of enhancers, 89% of known B-cell and 71% of known T-cell chromatin annotated as predicted enhancers were present.

B Enrichment of ENCODE TF binding sites (TFBS) in chromatin states. The average profile is shown for each cluster of TFBSs (TFBS C1–C2) with the TFBS description given in Appendix Table S5.

C Enrichment of bidirectionally expressed RNA from all samples and chromatin states changed in CLL in comparison with published data sets. These included the enhancer catalogs from Vista, modifications from ENCODE, bidirectional RNA from FANTOM, and p300 sites from ENCODE. The majority of known enhancers from diverse tissues corresponded to our active 1–3 states. Inactivation of these states in CLL occurred mostly via the bivalent state.

D Chromatin feature maps at the *CREB3L2* for a CLL patient (CLL1) and an NBC donor (H7). An intragenic predicted enhancer region downstream of the TSS that became active in CLL is highlighted. Light gray depicts active chromatin region and dark gray the confined enhancer locus coinciding with an open chromatin region. For color coding of ChromHMM states, see panel (B).

E Same for the *FMOD* locus with two predicted enhancer regions with enhanced activity in CLL marked in gray.

© 2019 The Authors

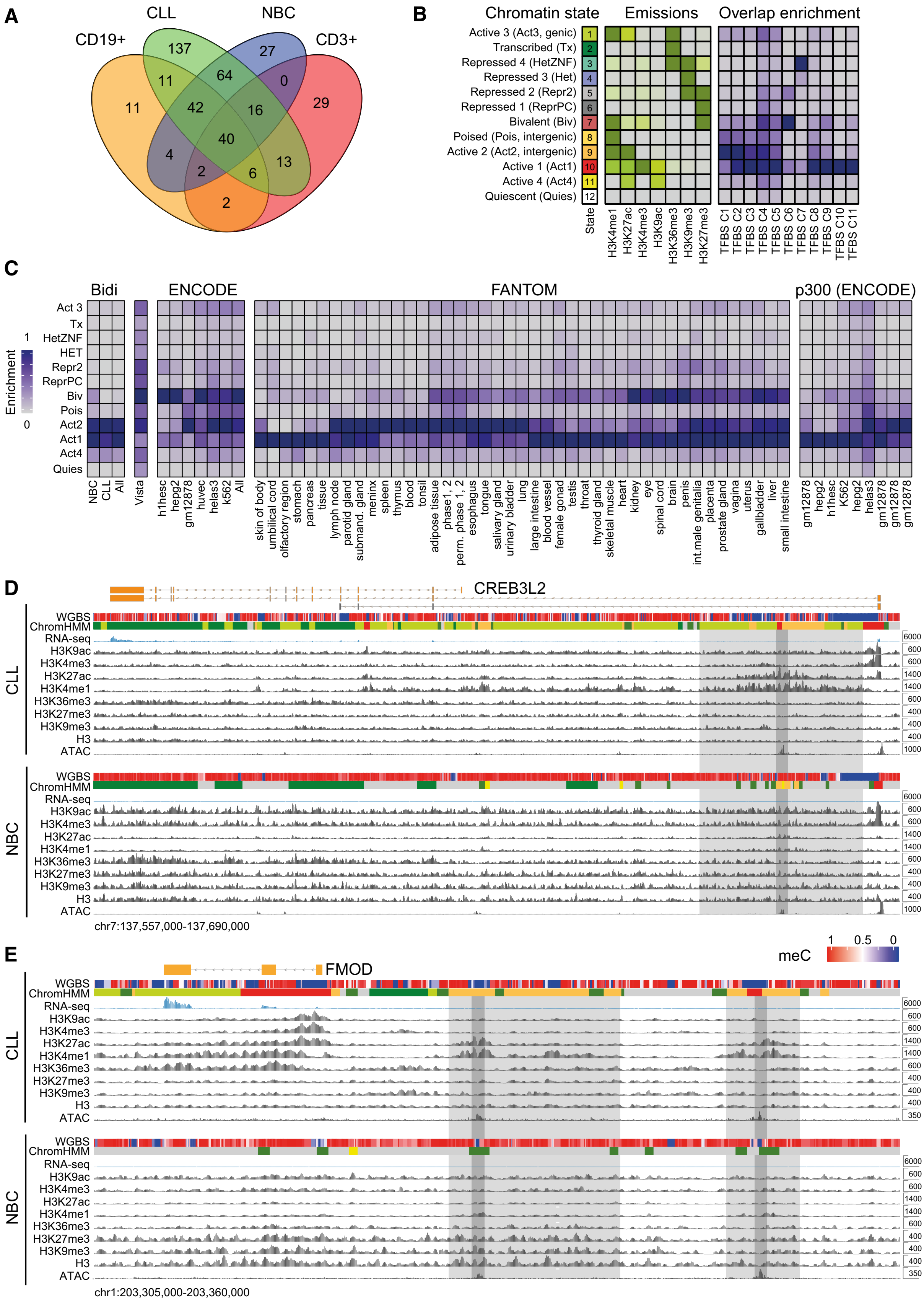

Figure EV4.

 © 2019 The Authors

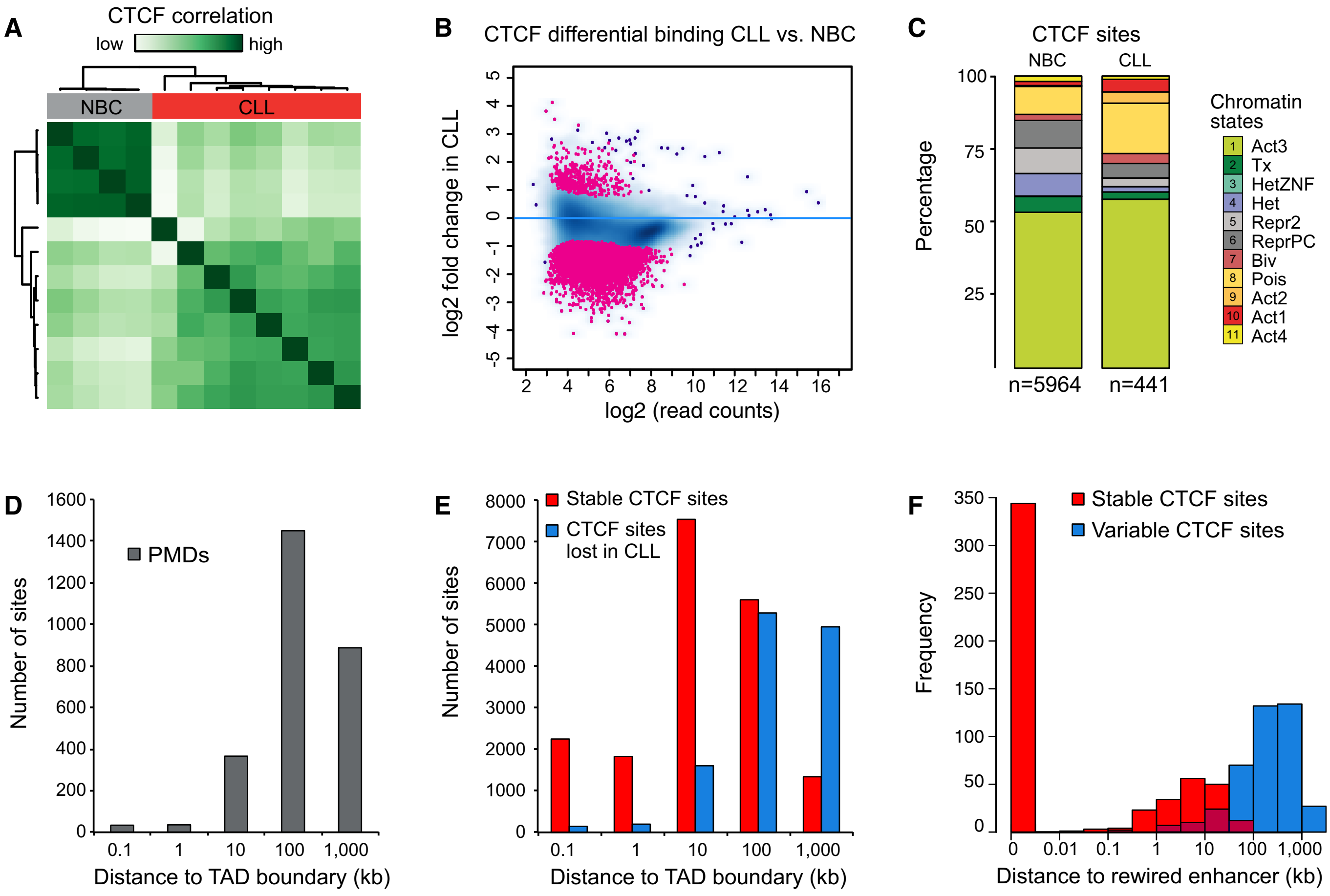

Figure EV5. Analysis of CTCF binding by ChIP-seq.

A Unsupervised clustering of CTCF ChIP-seq reads. The ChIP-seq signal separates NBC and CLL samples.

B MA plot of CTCF ChIP-seq regions with differentially occupied CTCF ChIP-seq regions marked in red. The plot showed a large-scale loss of CTCF binding activity in CLL.

C Chromatin state annotation of differential CTCF ChIP-seq binding sites.

D Histogram displaying the distance of PMD to the TAD boundaries reported previously for a lymphoblastoid cell line (Rao *et al*, 2014). Size distribution (average and std. dev) was 1,156 kb ± 125 kb (PMDs, this study) and 258 kb ± 20 kb [TADs (Rao *et al*, 2014)].

E Histogram displaying distance of CTCF sites to TAD boundaries for stable and lost CTCF sites.

F Histogram displaying the distance of lost and stable CTCF sites to enhancers that change their gene target in CLL.

© 2019 The Authors
