# Supplementary material for: Linking aberrant chromatin features in chronic lymphocytic leukemia to transcription factor networks
Source: Mol Syst Biol. 2019 May 22;15(5):e8339. doi: 10.15252/msb.20188339 (PMC6529931; doi:10.15252/msb.20188339)
Supplement: Supplementary file 16 — Dataet EV14 [file MSB-15-e8339-s016.zip › Dataset_EV14/README.txt]

The .cys file can be opened using Cytoscape https://cytoscape.org
